# Measuring entomological parameters before implementing a study on asymptomatic carriers of *Plasmodium falciparum* in the Zè District in southern Benin

**DOI:** 10.1186/s12936-023-04450-4

**Published:** 2023-01-21

**Authors:** Aziz Bouraima, Armel Djènontin, Yannelle Dossou, Lenucthadius Houessou, Christophe Soares, Montchédé Anato, Boris-Enock Zinsou, Célia Dechavanne, Jerome Clain, Achille Massougbodji, Gilles Cottrell

**Affiliations:** 1 Centre de Recherche Pour La Lutte Contre Les Maladies Infectieuses Tropicales (CReMIT), Université d’Abomey-Calavi (UAC), BP 526, Cotonou, Bénin; 2 Centre de Recherche Entomologique de Cotonou (CREC), 06 BP 2604, Cotonou, Bénin; 3 Institut de Recherche Clinique du Bénin (IRCB), 04 BP 1114, Cotonou, Bénin; 4 Université Paris Cité, IRD, MERIT, 75006 Paris, France; 5 Centre d’Etude Et de Recherche Sur Les Pathologies Associées À La Grossesse Et À L’Enfance (CERPAGE), Cotonou, Bénin

**Keywords:** Malaria, Transmission, Zè District, *Anopheles gambiae*, Insecticide resistance

## Abstract

**Background:**

The objective of this study was to estimate malaria transmission and insecticide resistance status in malaria vectors in Adjrako village from Zè District in Southern Benin. The present study was carried out prior to investigations on infectivity of blood from asymptomatic carriers of *Plasmodium falciparum* to malaria vector mosquitoes*.*

**Methods:**

Human landing collections (HLCs) were performed in Adjrako village during the rainy season (September—November 2021). In this village, host-seeking mosquitoes were collected during three nights per survey from 22:00 to 06:00 in six randomly selected houses. Malaria vectors were dissected in orders to determinate their parity. *Plasmodium falciparum* infection in malaria vectors was determined by qPCR and the entomological inoculation rate (EIR) was calculated. The World Health Organization (WHO) insecticide susceptibility test-kits were used to evaluate the susceptibility of *Anopheles gambiae *sensu lato (*s.l.*) to deltamethrin at 0.05% and bendiocarb at 0.1%.

**Results:**

A total of 3260 females of mosquitoes belonging to 4 genera (*Anopheles*, *Culex*, *Aedes* and *Mansonia*) were collected. Most of the mosquitoes collected were *An. gambiae *sensu lato (*s.l.*). The entomological inoculation rate (EIR) for the three collection months was 8.7 infective bites per person and the parity rate was 84%. Mortality rates of *An. gambiae s.l.* exposed to 0.05% deltamethrin and 0.1% bendiocarb were 18% and 96%, respectively, indicating that this vector population was resistant to deltamethrin and possibly resistant to bendiocarb in the study area.

**Conclusion:**

This study showed that malaria transmission is effective in the study area and that *An. gambiae s.l.* is the main malaria vector. The entomological parameters indicate this study area is potentially favourable for investigations on *P. falciparum* asymptomatic carriers.

**Supplementary Information:**

The online version contains supplementary material available at 10.1186/s12936-023-04450-4.

## Background

Despite intensified interventions and recent control efforts, malaria remains a public health problem. In the WHO African Region, malaria cases and deaths increased in 2021 from 213 to 228 million, and from 534,000 to 602,000, respectively [1]. In Benin, malaria is the first cause of hospitalization and healthcare use and represents 46.8% of the reasons for consultation in the population [2]. The incidence and mortality of malaria in the Beninese population are 14.6% and 0.9‰, respectively [3].

Malaria is caused by *Plasmodium falciparum* transmitted by female *Anopheles*. Out of the 476 *Anopheles* species, approximately 70 are competent of transmitting malaria [4]. In tropical Africa, the main vectors are *Anopheles gambiae*, *Anopheles arabiensis*, *Anopheles funestus*, *Anopheles nili*, *Anopheles moucheti*, *Anopheles melas* and *Anopheles merus* [5]. These malaria vectors belong to species complexes or groups of species [6] and exploit a wide variety of water collections as breeding sites, including residual pools of sunny stagnant surfaces, vegetated pools and brackish waters [7]. In Benin, the dominant vectors of *P. falciparum* are *An. gambiae *sensu lato (*s.l.*), *An. funestus* [8] and *An. nili* [9].

Malaria transmission is most active during the rainy season, which is the most favourable period for vector proliferation. In Africa, transmission is generally high and the entomological inoculation rates (EIR) are frequently greater than 100 infective bites/person/year and can reach 1000 infective bites/person/year. Transmission is heterogeneous and varies according to the areas [10, 11]. In Benin, malaria transmission varies considerably from one region to another and is higher in rural than in urban areas. From south to north, transmission is high in a Guinean climate (June–August); low in a sub-equatorial climate (March–August; October–November); continuous moderate in a Sudano-Sahelian climate and perennial in a Sudanese climate. The entomological inoculation rate varies between 0 and 8 bites per person per night [12–14]. Insecticide resistance in malaria vectors represents a concern for the success of malaria control programmes [1]. These vectors are becoming more and more resistant to pyrethroids and other classes of insecticides [15–17]. Previous studies in several regions have reported the emergence and expansion of insecticide resistance in malaria vectors, particularly to bendiocarb and deltamethrin [18–21].

Several strategies to control malaria have been developed, including vector control, which is the main means of preventing malaria transmission. According to World Health Organization (WHO) recommendations, this control is based mainly on the use of insecticide-treated nets (ITNs) and indoor residual spraying (IRS) [1]. ITNs and IRS have been shown to be effective in reducing malaria-related morbidity and mortality [22, 23]. Over the past decades, worldwide and regional efforts have led to a sharp decline in malaria-related morbidity and mortality. Indeed, between 2001 and 2013, the dramatic scale-up of malaria control interventions contributed to a 47% reduction in malaria mortality rates in the world, averting an estimated 4.3 million deaths [24].

In order to continue to successfully control malaria, new strategies must be developed to interrupt transmission in parasite-carrying subjects. For example, reducing transmission from humans to mosquitoes could greatly improve the efforts of control programmes. Thus, before implementing a study on the infectivity of *P. falciparum* asymptomatic carriers for malaria vectors, it is important to conduct an entomological study to determine the composition of mosquito populations, to assess the resistance status of *An. gambiae* to insecticides, and to estimate malaria transmission in the selected area.

## Methods

Collections of larvae and adult mosquitoes were carried out in this study**.**

### Study area

**Fig. 1:**
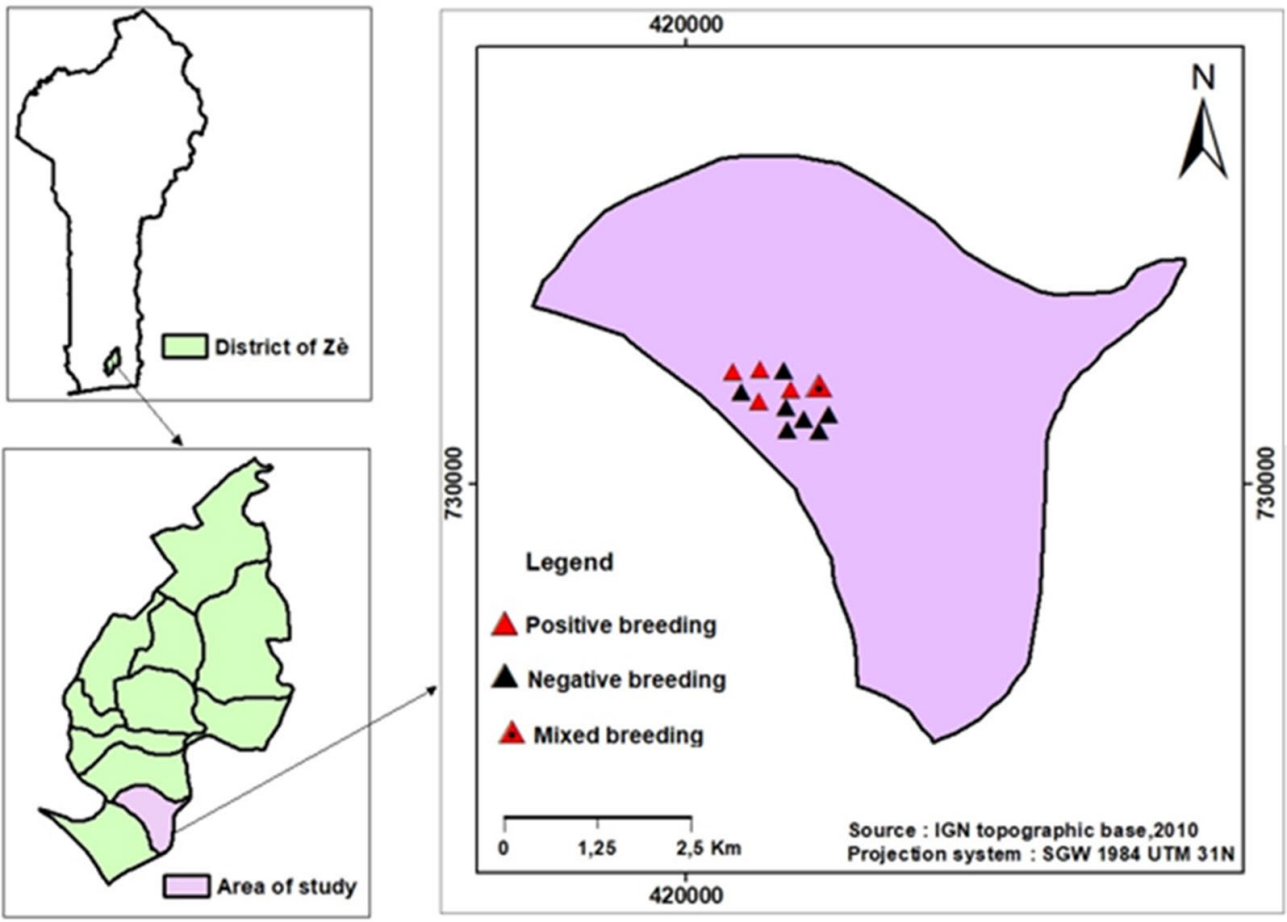
Map of study area

The study was carried out in Adjrako, a village in the arrondissement of Yokpo in the Zê District. Adjrako village was selected based on accessibility during the rainy season and the proximity with the laboratory research. The estimated population of this village is 800 inhabitants. The Zê District is located in the North-East of the Atlantic department (between 6°32ʹ and 6°87ʹ North latitude and between 2°13ʹ and 2°36ʹ East longitude) (Fig. 1). The average annual rainfall is 1257.95 mm and the average temperature is 27.4 °C. The climate of the Zè District is characterized by the 4 seasons: 2 rainy seasons from April to July and from September to November and 2 dry seasons from December to March and in August. The hydrographic network of the district of Zè is not very dense. The presence of shallows and water courses in this district provides facilities for the cultivation of corn, cassava, pineapple and peanuts. Long-lasting insecticidal nets (LLINs) represent the main malaria vector control intervention in Adjrako; LLINs are freely distributed by the government every 3 years [25].

### Adult mosquito collection

Three entomological surveys were conducted in the study area. Surveys were carried out from September to November 2021 corresponding to the short rainy season. At each survey, we performed human landing collections (HLCs) in the Adjrako village according to the method used by Coffinet et al. [26]. Host-seeking mosquitoes were collected during 3 consecutive nights per survey from 22:00 to 06:00 inside and outside in six randomly selected houses. Mosquito collection houses were not varied throughout the study. Mosquitoes were collected by 12 collectors (composed exclusively of males and at least 18 years of age) at each survey. Each selected house had one mosquito collector indoors and one collector outdoors. Each night of collection, one technician from the Centre for Research in Entomology of Cotonou (CREC) assisted by one local supervisor supervised the mosquito collections in this village, to ensure that they were performed properly. Mosquitoes collected were stored individually in tubes and closed with cotton wool and then kept in small bags by time slot and collection point. All mosquitoes collected were returned to the laboratory, examined under a binocular loupe, and identified using identification keys [27]. It is an illustrated key presenting, in the form of drawings, the characters corresponding to each species of mosquito.

### *Anopheles* vector processing

After morphological identification, only *An. gambiae s.l.* and *An. funestus* mosquitoes were kept and were dissected in order to determinate their parity according to the appearance of the ovarian tracheoles. Dissection of mosquito ovaries was performed in distilled water with appropriate forceps. After dissection, the status of the tracheoles on the ovaries was examined under a microscope. In nulliparous females, the tracheoles were found to be rolled up and unrolled in parous females [28]. The head-thorax complex of each mosquito was cut and stored individually in 1.5 ml eppendorf tubes for detection for infection (vectors carrying *P. falciparum* sporozoites) by the quantitative PCR (qPCR). The mosquito carcasses (wings, legs and abdomen) were conserved in silica gel and stored at − 20 °C.

### Detection of *Plasmodium* in malaria vectors

The head-thorax portion of each *Anopheles* was ground in a blocking buffer [29]. Genomic DNA was extracted from the head-thorax grindings of each mosquito using the DNeasy*H* Blood & Tissue kit (Qiagen) as recommended by the manufacturer. This procedure has been validated for the detection of *Plasmodium* in mosquitoes [30]. A SYBR Green real-time PCR restriction fragment length polymorphism assay (cytb-qPCR) targeting the cytochrome b gene of the human *Plasmodium* species was used [31]. To distinguish between *P. falciparum* and non-*P. falciparum* species, *P. falciparum* specific primer was used. For the cytochrome *b* gene (*cytb*) qPCR assay, the primers used were:

*Pf_cytb*_ TTGGTGCTAGAGATTATTCTGTTCCT) and *Pf_cytb*_ GGAGCTGTAATCATAATGTGTTCGTC) [32].

The PCR reaction was carried out in a final volume of 20 μl, containing 5 μl of extracted DNA, 0.25 μM of each primer. A fluorescence reading for each sample was taken. Tested DNA samples were scored *P. falciparum* positive when *pf_cytb* qPCR gave a Ct (cycle threshold) ≤ 22.35 with a melt curve and temperature similar to corresponding positive controls. All amplification and melt curves were individually inspected by eye.

### Characteristics of breeding sites

In addition to collecting adult mosquitoes, we collected larvae and characterized their breeding sites as follows. All breeding sites visited and explored during this survey were geo-referenced using Geographical Positioning System (GPS) device integrated in smartphone. The mapping of these sites was carried out using ArcGIS version 10.3 software. All breeding sites with at least one *Anopheles gambiae s.l.* larva were considered positive and the others negative. For each larval site, the type of breeding site (puddle, floor tanks, plastic containers, village pump) was noted and its area classified as: (i) small area (< 1 m^2^); (ii) medium area (1–5 m^2^) or (iii) large area (> 5 m^2^). The nature of sites in which larvae were collected was noted as: polluted, unpolluted, permanent, semi-permanent, temporary, sunny, not sunny or partially sunny.

### Larvae and pupae collection and density determination

Larval surveys were carried out in the village and its surroundings. Collections of water likely to harbor pre-imaginal stages of mosquitoes were prospected according to the method used by Coffinet et al. [26]. Larvae and pupae were collected once during the study period from several breeding sites in the village using the dipping method [33]. Before sampling, a waiting period of few minutes was observed to allow mosquito larvae, if there were any, to rise to the surface of the water. The mosquito larvae were morphologically identified to the genus level using reference keys [34]. Larvae and pupae collected were kept in plastic containers with a little water. The average larval and pupal densities (for 3 dips) obtained in each site were registered. The larvae and pupae were reared in the insectarium of Center for Research in Entomology of Cotonou (CREC) in a room with a relative humidity between 70 and 80% and a temperature between 25 and 30 °C. Photoperiod was of 12 h of light and 12 h of darkness.

### Bioassays

Female mosquitoes aged 2–5 days obtained from larvae and pupae and morphologically identified as *An. gambiae s.l.* were exposed to doses of deltamethrin (0.05%) and bendiocarb (0.1%) as described in the standard WHO testing protocol [35]. For each insecticide, 4 replicates of 25 mosquitoes were exposed for 1 h to the treated papers. As a control, 4 others batches of 25 mosquitoes were exposed to untreated papers. The number of mosquitoes knocked down by the insecticide after 60 min of exposure was registered. After 60 min of exposure, the mosquitoes were fed with a 10% glucose solution and observed for 24 h. After 24 h of observation, mortality rates were determined. The resistance status of mosquitoes in the study area was determined according to WHO criteria [35].

### Data analysis

Human biting rate (HBR) was expressed as the number of bites/person per time unit. The sporozoite rate (SR) was calculated as the proportion of mosquitoes found positive by qPCR out of the total tested. The Entomological Inoculation Rate (EIR) was calculated according to the formula: EIR = HBR x SR. The parity rate was expressed as the percentage of parous mosquitoes out of the total dissected. The Pearson correlation coefficient test was used to determine the linear correlation between longevity and malaria vector transmission using Statistica version 7.0.61.0 EN. Mosquito susceptibility and resistance to insecticides was defined according to WHO criteria [35]: mortality > 98% indicates a susceptible population; mortality between 90 and 98% suspected resistance and mortality < 90% indicates a resistant population.

## Results

### Culicidae fauna

During this study, 3260 adult mosquitoes were captured inside and outside of houses. Four genera (*Anopheles*, *Culex*, *Aedes* and *Mansonia*) with seven species of mosquitoes (*An. gambiae s.l*, *An. funestus*, *Anopheles ziemanni*, *Culex gr decens*, *Culex quinquefasciatus*, *Culex annulioris*, *Aedes aegypti* and *Mansonia africana*) were identified in the area (Table 1). *Anopheles gambiae s.l.* and *An. funestus* are species complexes that are each comprised of several different species that are morphologically indistinguishable. The genera *Culex*, *Aedes* and *Mansonia* constituted 92.85% (3027/3260), 0.61% (20/3260) and 3.59% (117/3260) of mosquitoes captured respectively. *Anopheles* species were weakly represented with 2.85% (93/3260) for *An. gambiae s.l.*, 0.06% (2/3260) for *An. funestus* and 0.03% (1/3260) for *An. ziemanni. Anopheles gambiae* was the most abundant *Anopheles* species and constituted 97% (93/96) of the *Anopheles* population captured.

**Table 1:** Diversity of mosquito species in Adjrako during the three surveys

Mosquito species	Survey 1 September 2021	Survey 2 October 2021	Survey 3 November 2021	Total (%)
***Anopheles gambiae s.l***	**71**	**21**	**1**	**93 (2.85)**
***Anopheles funestus***	**2**	**0**	**0**	**2 (0.06)**
*Anopheles ziemanni*	0	1	0	1 (0.03)
*Mansonia africana*	90	18	9	117 (3.59)
*Culex gr decens*	2521	387	96	3004 (92.15)
*Culex quinquefasciatus*	20	0	0	20 (0.61)
*Culex annulioris*	0	3	0	3 (0.09
*Aedes aegypti*	11	8	1	20 (0.61)
**Total**	**2715**	**438**	**107**	**3260 (100)**

The malaria vectors and all its data are in bold. As well as the total number of all mosquitoes that were captured in this study.

### Human biting rate, longevity, entomological inoculation rate and *Plasmodium falciparum* infection

**Fig. 2:**
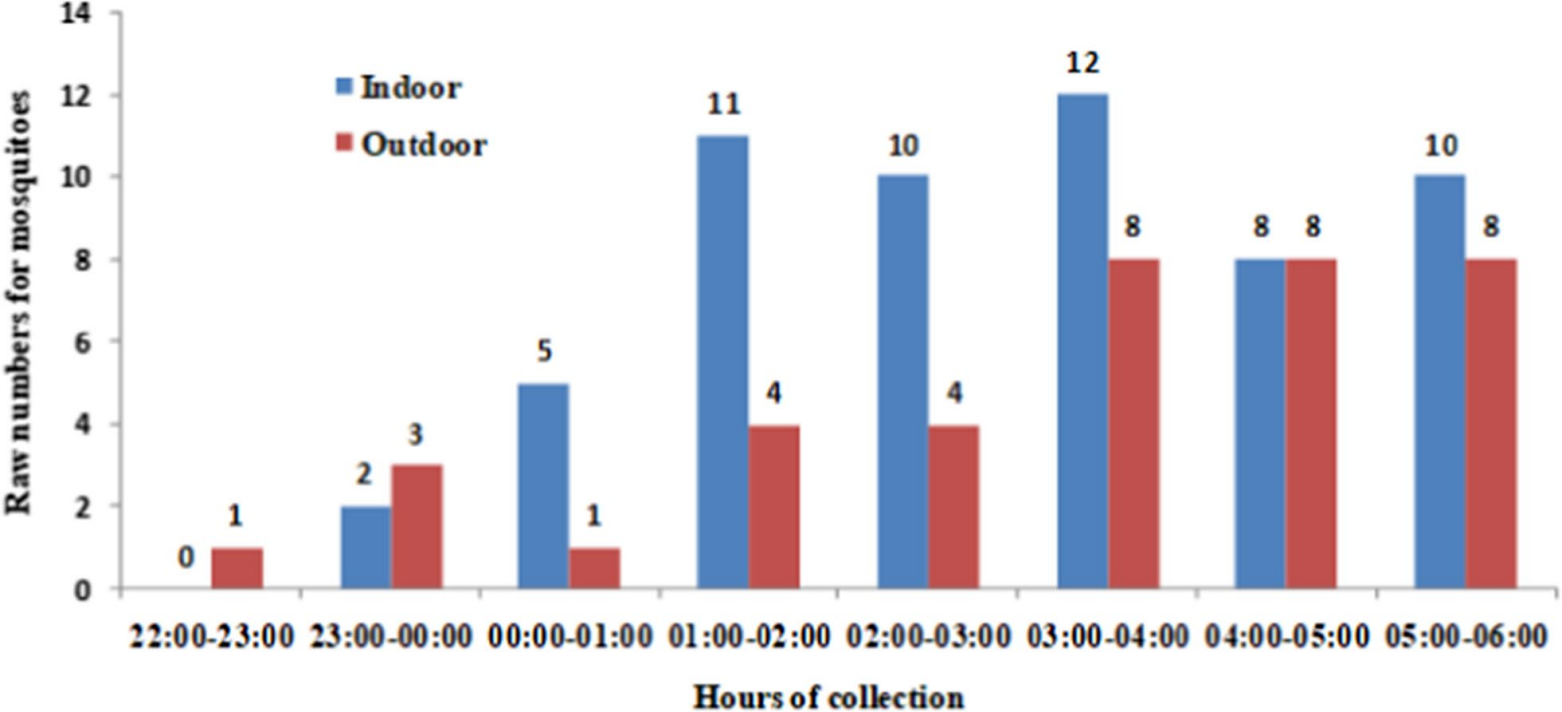
Malaria vectors biting rhythm indoor and outdoor

In this study, the majority of *Anopheles* mosquitoes were collected inside houses (Table 2). Malaria vectors bites occurred principally from 23:00 to 06:00 both indoor and outdoor (Fig. 2). The peak of human biting rate was between 03:00 and 04:00 and was observed inside the houses. Human biting rate was 96.7 bites per person were recorded inside houses and 61.7 outside for three months of collection. The data concerning the number of mosquitoes captured per time slot, the human biting rate, the number tested and positive for qPCR, the entomological inoculation rate were showed in the Additional file 1.

**Table 2:** Longevity and *Plasmodium falciparum* infection in malaria vectors in Zè District during the study

	Number malaria vectors tested	Malaria vectors longevity		*P. falciparum* infection in malaria vectors	
		Number malaria vectors parous	% [95% CI]	Number malaria vectors infected	% [95% CI]
Indoor	58	49	85 [75–94]	8	14 [5–23]
Outdoor	37	31	84 [72–96]	2	5 [0–13]
Total	**95**	**80**	**84 [77–94]**	**10**	**11 **[4–17]

The overall parity rate of the collected malaria vectors was 84% (Table 2). All malaria vectors captured were tested for infective *P. falciparum* sporozoites by qPCR. For *An. gambiae s.l.* the SR was 10.75% (10 positive head-thoraces out of 93 tested) whereas neither of the two *An. funestus* was positive (SR = 0%). The overall entomological inoculation rate (EIR) for the three collection months (period from September to November corresponding to the short rainy season) was 8.7 infective bites per person. Statistical analysis showed that there was a significantly positive correlation between vector longevity and transmission in the study area (r = 0.07 and p < 0.05).

### Characteristics of breeding sites

The results on the characteristics of the breeding sites are presented in Table 3. A total of 12 breeding larval sites were geo-referenced. Among these sites, 5 contained at least one *Anopheles* larva. The other sites without *Anopheles* were considered as negative sites. Most of the larval breeding habitat (91.66% n = 11/12) were temporary sites composed mainly of puddles (75% n = 9/12) that were formed after the rains. Concerning the level of pollution, 9 of the sites (75% n = 9/12) were polluted and the majority of the geo-referenced sites (83.33% n = 10/12) had an average surface area. The mosquito larvae observed in the sites were *Culex* larvae, *Anopheles* larvae and a mixture of the two genera. The average larval and pupal densities calculated after 3 dips in each of the *Anopheles* positive sites were highly variable (Table 3). The minimum larval density was 1 larva per dip and the maximum was 23 larvae per dip. As for the pupal density, it was a minimum of 1 pupa per dip and a maximum of 13 per dip.

**Table 3:** Characteristics of larval breeding sites

Breeding sites	Characteristics	Mosquito larvae	Larval density			Pupal density		
			Minimum	Maximum	Average	Minimum	Maximum	Average
**1**	Temporary							
	Puddle							
	Medium area	*Culex*	> 100	> 100	> 100	21	34	27
	Polluted							
	Sunny							
**2**	Temporary							
	Plastic container							
	Small area	*Culex*	3	14	8	1	4	2
	Polluted							
	Partly sunny							
**3**	Temporary							
	Puddle							
	Medium area	***Anopheles***	**17**	**23**	**20**	**3**	**11**	**8**
	Not polluted							
	Sunny							
**4**	Temporary							
	Puddle							
	Medium area	*Culex*	21	37	28	7	14	11
	Polluted							
	Sunny							
**5**	Permanent							
	Floor tanks							
	Large area	*Culex*	16	24	20	1	5	3
	Polluted							
	Partly sunny							
**6**	Temporary							
	Puddle							
	Medium area	***Anopheles***	**8**	**11**	**9**	**3**	**8**	**5**
	Polluted							
	Partly sunny							
**7**	Temporary							
	Puddle							
	Medium area	*Culex*	> 100	> 100	> 100	23	31	27
	Polluted							
	Sunny							
**8**	Temporary							
	Puddle							
	Medium area	***Anopheles***	**13**	**17**	**15**	**1**	**2**	**1**
	Polluted							
	Sunny							
**9**	Temporary							
	Puddle	*Culex*	13	21	18	4	11	7
	Medium area	*&*						
	Polluted	***Anopheles***	**1**	**4**	**3**	**1**	**2**	**1**
	Sunny							
**10**	Temporary							
	Puddle							
	Medium area	*Culex*	29	47	37	11	21	16
	Polluted							
	Sunny							
**11**	Temporary							
	Puddle							
	Medium area	*Culex*	17	24	21	4	11	7
	Polluted							
	Sunny							
**12**	Temporary							
	Village pump							
	Medium area	***Anopheles***	**5**	**11**	**8**	**1**	**2**	**1**
	Not polluted							
	Sunny							

The larval and pupal densities of malaria vectors (Anopheles) collected at the breeding sites are shown in bold.

### Vector resistance status

**Fig. 3:**
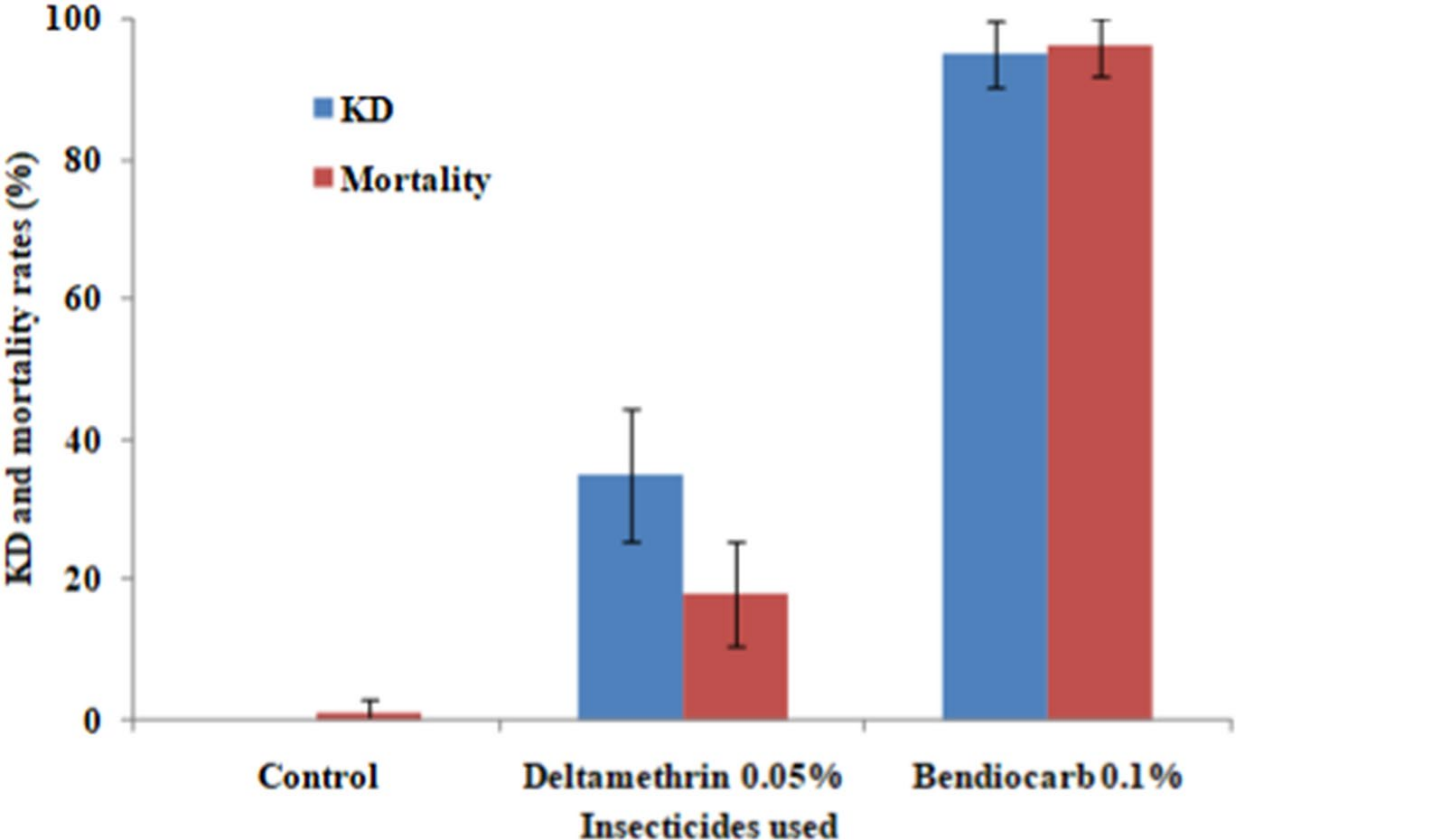
Knock-down and mortality rate of female *Anopheles gambiae s.l.* exposed to insecticide

The results of the susceptibility tests showed the knock down (KD) rate of *Anopheles* collected in the study village after 60 min exposure to 0.05% deltamethrin was 35%, and the mortality rate 24 h after exposure was 18%. These susceptibility tests showed that malaria vector population was resistant to deltamethrin. In contrast, after exposure to 0.1% bendiocarb, the KD and mortality rates were 95% and 96%, respectively (Fig. 3). These results show a possible resistance to bendiocarb in the study area.

### Discussion and conclusion

Four mosquito genera and seven species were collected during entomological surveys carried out in the Adjrako village. The *An. gambiae* complex was the main malaria vector with 8.7 infected bites per person for the period from September to November. This malaria vector was resistant to deltamethrin.

Seven species collected during the present study are lower than that recorded by Djènontin et al. [8] in Ouidah-Kpomasse-Tori health zone in southern Benin and Yadouléton et al. [36] in the District of Corpargo in the North-East of Benin, but is consistent to those recorded by Djègbè et al. [37] in Lélé, in southern Benin. Although Ouidah-Kpomasse-Tori health zone and the district of Zè are located on the same plateau of Allada, lower species numbers were recorded in the present study. These results could be explained by large surface area of the study conducted by Djènontin et al. [8]. Indeed, these authors have carried entomological surveys in 28 villages in 3 districts, in contrast with the present study carried out in 1 district. This suggested a probable heterogeneity in mosquitoes distribution on the plateau of Allada. Similarly, this difference observed at the species level with these authors could be related to the identification method used. Only morphological identification with a taxonomic key was used in this study, while other authors had also used molecular method; a more advanced method in the determination of species. In the same, the lower species number could be explained by the relatively short mosquitoes collection period.

Regarding the species richness of the different malaria vectors collected in this study, two potential vectors *An. gambiae s.l.* and *An. funestus* were collected. *Anopheles gambiae s.l.* was the most abundant species of the anopheline fauna and the majority was captured inside the houses, confirming the endophagous nature of this species. The density of *An. gambiae s.l.* obtained in this study was very low compared to that obtained by Djègbè et al. [37]; this low density could be related to the hydro-geographical factors of the study area. The study by Djègbè et al. [37] was conducted in a rice-growing area. As reported in several studies, rice fields are a favourable environment for the larval development of this species [38, 39]. The number of *An. funestus* vectors caught in this study was very low. This result suggests that breeding sites for this species are rare or scarce in the study area as *An. funestus* species prefer shaded sites with vegetative broods [34]. Although this species has a nocturnal biting behaviour especially in the second part of the night [40, 41], it could shift its biting time, as was reported by others [42, 43] who had observed females of this species biting at the beginning of the day. The continuation of the collection of mosquitoes until 09.00 in the morning could give more information on the density of this species.

The high rate of *Culex* genus observed in this study could be explained by the abundance of polluted breeding sites that were favourable for the proliferation of *Culex* larvae [44]. These observations corroborate those made by the studies that have shown that mosquito species belonging to the *Culicinae* have strong adaptive capacities allowing them to develop in polluted environments [45, 46]. Similar observations have been made by Koumba et al. [47], who showed that immature stages of the genus *Culex* are found in the larval sites rich in organic matter.

The larval surveys carried out in the context of this study showed that the study area has a diversity of larval sites. The majority of *Anopheles* larval sites identified in this study were temporary, unpolluted and sunny puddles sites. This result corroborates those of some authors who have shown that *An. gambiae s.l.* females prefer to lay their eggs in sunny water collections devoid of vegetation [39, 48]. However, the very low number of positive *Anopheles* breeding sites identified is probably associated to the heavy rains observed in the study area a few days before the larval surveys. These rains would have washed away several breeding grounds which would be favourable to the development of *Anopheles* larvae. The cohabitation of *Anopheles* and *Culex* larvae in the same breeding site suggests that *Culex* is able to survive in both clean and polluted habitats.

Populations of *An. gambiae*, the main malaria vector collected in this village, have developed resistance to deltamethrin and possibly resistance to bendiocarb. The results obtained in this study corroborate those of Djègbè et al. [21]*,* who observed deltamethrin resistance in the same district, but with a mortality rate of 86%. The massive and sometimes abusive use over the years of household insecticides, such as aerosol cans, coils and especially the emergence of other insecticidal chemicals used against mosquito bites could certainly have contributed in one way or another to the selection of this resistance. Resistance of *An. gambiae s.l.* to deltamethrin had been previously reported in Benin [19, 20, 37].

The possibly resistance of mosquitoes to bendiocarb observed in our study is believed to be due to the massive use of pesticides in the agricultural environment in the Zè district. Indeed, pineapple cultivation is the most recurrent product in agricultural production in Zè district and farmers often resort to the use of chemicals, such as insecticides, herbicides or fungicides for various reasons [49–51].

Malaria transmission in the study area seems to be carried out mainly by *Anopheles gambiae s.l.,* the major vector of malaria in Benin [8, 50, 52, 53]. The qPCR results confirmed that no infected *An. funestus* was found, but in view of the low number of *An. funestus* collected this may not be conclusive. The infectivity of this vector has been observed in southern Benin by several studies [8, 54, 55].

Determination of plasmodial infection by qPCR in mosquito heads and thorax showed that 11% of mosquitoes were infected. Seen that PCR-based methods detect *Plasmodium* in stages other than sporozoites, sporozoite index observed in the present study could be an overestimate [56].

The overall EIR for this study was 8.7 infecting bites per person for the period from September to November (corresponding to the short rainy season). The transmission was highest in September which corresponds to the beginning of the short rainy season with EIR 6.1 infecting bites per person and zero in November which marks the end of this season. This suggests that, the population of Zè is much more exposed to *P. falciparum* transmission during rainy seasons when mosquitoes are abundant. These results corroborate those obtained in a northern region which showed that transmission is very high in the rainy season than in the dry season [36]. This information is likely to favour the carriage of *Plasmodium* in the Adjrako population especially in the rainy season. Asymptomatic carriage of malaria parasites being common in all malaria-endemic areas [57], so the village of Adjrako is a very favourable location for the implementation of the study on the infectivity of asymptomatic carriers of *P. falciparum*.

This transversal study on entomological parameters provides a data set on malaria transmission in the village of Adjrako in the district of Zè. However, the three months data collection periods corresponding to the short rainy season are insufficient to assess culicidian diversity and malaria transmission in this district. A longitudinal study would be necessary to measure the variability of malaria vectors and the physico-chemical and biological parameters of the larval sites over all seasons of the year. Despite its limits, the data from this study are importance for the study for which it was collected. Indeed, the main vector of malaria in the study area is known and this can orientate the tests to be carried out within the framework of the study on asymptomatic carriers which will be set up.

## Supplementary Information

**Additional file 1**: **Table S1**. Hourly distribution of Human biting rate and Entomological Inoculation Rate.

## Data Availability

The raw data used in this study are available from the corresponding author upon reasonable request.

## Abbreviations

CREC: Centre de recherche entomologique de cotonou
HLCs: Human landing collections
Cytb: Cytochrome b
DNA: Deoxyribonucleic acid
EIR: Entomological inoculation rate
GPS: Geographical positioning system
IRS: Indoor residual spraying
ITNs: Insecticide-treated nets
KD: Knock down
PCR: Polymerase chain reaction
Pf_cytb: *Plasmodium falciparum* cytochrome b
qPCR: Quantitative polymerase chain reaction
Ct: Cycle threshold
SYBR: Synergy brands
WHO: World Health Organization
