# Supplementary material for: Measuring entomological parameters before implementing a study on asymptomatic carriers of Plasmodium falciparum in the Zè District in southern Benin
Source: Malar J. 2023 Jan 21;22:24. doi: 10.1186/s12936-023-04450-4 (PMC9862539; doi:10.1186/s12936-023-04450-4)
Supplement: Supplementary file 1 — Additional file 1: Table S1. Hourly distribution of Human biting rate and Entomological Inoculation Rate. [file 12936_2023_4450_MOESM1_ESM.docx]

**TableS1: Hourly distribution of Human biting rate and Entomological Inoculation Rate**

|  |  |  |  |  |  |
| --- | --- | --- | --- | --- | --- |
|  | **Malaria Vectors** | | | | |
| **Hours** | **Number** | **Human biting rate** | **Number positive by qPCR** | **Sporozoite rate** | **EIR** |
| 22:00-23:00 | 1 | 0.01 | 0 | 0.00 | 0.00 |
| 23:00-00:00 | 5 | 0.05 | **1** | 0.20 | 0.01 |
| 00:00-01:00 | 6 | 0.06 | **1** | 0.17 | 0.01 |
| 01:00-02:00 | 15 | 0.14 | **2** | 0.13 | 0.02 |
| 02:00-03:00 | 14 | 0.13 | 0 | 0.00 | 0.00 |
| 03:00-04:00 | 20 | 0.19 | **2** | 0.10 | 0.02 |
| 04:00-05:00 | 16 | 0.15 | 0 | 0.00 | 0.00 |
| 05:00-06:00 | 18 | 0.17 | **4** | 0.22 | 0.04 |
| **Total** | **95** | **0.88** | **10** | **0.11** | **0.09** |
|  |  |  |  |  |  |
